# Infigratinib Reduces Fibroblast Growth Factor 23 (FGF23) and Increases Blood Phosphate in Tumor‐Induced Osteomalacia

**DOI:** 10.1002/jbm4.10661

**Published:** 2022-07-22

**Authors:** Iris R. Hartley, Kelly L. Roszko, Xiaobai Li, Karen Pozo, Jamie Streit, Jaydira del Rivero, M. Teresa Magone, Michaele R. Smith, Roo Vold, Carl L. Dambkowski, Michael T. Collins, Rachel I. Gafni

**Affiliations:** ^1^ National Institute of Dental and Craniofacial Research, NIH Bethesda MD USA; ^2^ Biostatistics and Clinical Epidemiology, Clinical Center, NIH Bethesda MD USA; ^3^ National Cancer Institute, NIH Bethesda MD USA; ^4^ Ophthalmology Consult Services Section National Eye Institute (NEI) Bethesda MD USA; ^5^ Rehabilitation Medicine Department, Clinical Center, National Institutes of Health Bethesda MD USA; ^6^ QED Therapeutics San Francisco CA USA

**Keywords:** OSTEOMALACIA AND RICKETS, CLINICAL TRIALS, TUMOR‐INDUCED BONE DISEASE, THERAPEUTICS: OTHER, DISORDERS OF CALCIUM/PHOSPHATE METABOLISM: OTHER

## Abstract

Tumor‐induced osteomalacia (TIO) is a rare paraneoplastic syndrome caused by ectopic production of fibroblast growth factor 23 (FGF23) by phosphaturic mesenchymal tumors (PMTs). Acting on renal tubule cells, excess FGF23 decreases phosphate reabsorption and 1,25‐dihydroxy‐vitamin D (1,25D) production, leading to hypophosphatemia, impaired bone mineralization, pain, and fractures. Fibronectin 1‐fibroblast growth factor receptor 1 (FN1‐FGFR1) gene fusions have been identified as possible drivers in up to 40% of resected PMTs. Based on the presumptive role of FGFR1 signaling by chimeric FN1‐FGFR1 proteins, the effectiveness of infigratinib, a FGFR1‐3 tyrosine kinase inhibitor, was studied in an open‐label, single‐center, phase 2 trial. The primary endpoint was persistent normalization of blood phosphate and FGF23 after discontinuation. Four adults with TIO (two nonlocalized, two nonresectable PMTs) were treated with daily infigratinib for up to 24 weeks. All patients had a favorable biochemical response that included reduction in intact FGF23, and normalization of blood phosphate and 1,25D. However, these effects disappeared after drug discontinuation with biochemistries returning to baseline; no patients entered biochemical remission. In the two patients with identifiable tumors, 68Gallium (68Ga)‐DOTATATE and 18Fluoride (18F)‐Fluorodeoxyglucose (FDG) PET/CT scans showed a decrease in PMT activity without change in tumor size. Patients experienced mild to moderate, treatment‐related, dose‐limiting adverse events (AEs), but no serious AEs. Three patients had dose interruptions due to AEs; one patient continued on a low dose for the entire 24 weeks and one patient stopped therapy at 17 weeks due to an AE. The study closed early due to a failure to meet the primary endpoint and a higher‐than‐expected incidence of ocular AEs. Infigratinib treatment lowered FGF23, increased blood phosphate, and suppressed PMT activity, confirming the role of FGFR signaling in PMT pathogenesis. However, treatment‐related AEs at efficacy doses and disease persistence on discontinuation support restricting the use of infigratinib to patients with life‐limiting metastatic PMTs. © 2022 The Authors. *JBMR Plus* published by Wiley Periodicals LLC on behalf of American Society for Bone and Mineral Research. This article has been contributed to by U.S. Government employees and their work is in the public domain in the USA.

## Introduction

Tumor‐induced osteomalacia (TIO) is a rare paraneoplastic syndrome that presents clinically with pain, weakness, and fractures. It is caused by ectopic fibroblast growth factor 23 (FGF23) secretion by phosphaturic mesenchymal tumors (PMTs).^(^ ^1^, ^2^ ^)^ The primary site of FGF23 action is the proximal renal tubules, where, by signaling through fibroblast growth factor receptor 1 (FGFR1) and the coreceptor KLOTHO, it inhibits renal phosphate reabsorption and 1,25‐dihydroxy‐vitamin D (1,25D) production. The net result is chronic hypophosphatemia,^(^ ^3^, ^4^ ^)^ leading to rickets in children and osteomalacia in adults, and associated muscle weakness, bone pain, deformity, and fractures.^(^ ^4^ ^)^

PMTs are typically solitary, benign lesions that are often difficult to locate. When the tumor is found, complete surgical resection is usually curative. Some patients with TIO are not amenable to surgery due to tumor nonlocalization, significant surgical risk, distant or local metastases, or patient preference. If surgery is not possible, other tumor‐directed therapies, such as cryoablation, radiofrequency ablation, or radiotherapy have been used, although long‐term efficacy from these approaches is lacking.^(^ ^5^, ^6^, ^7^ ^)^

Conventional medical therapy, with either a combination of phosphate and calcitriol, or burosumab, an FGF23‐blocking monoclonal antibody, effectively treats the consequences of hypophosphatemia, but does not target the tumor directly.^(^ ^8^, ^9^ ^)^ With current medical treatment options, there remains a risk of continued tumor growth or tumor metastasis.^(^ ^10^, ^11^ ^)^

Chromosomal translocations resulting in a fibronectin 1‐FGFR1 (FN1/FGFR1) fusion gene have been identified in approximately 40% of resected PMTs.^(^ ^12^ ^)^ The fusion protein presumably includes the extracellular fibronectin autodimerization domain, and the FGFR1 ligand‐binding, transmembrane, and intracellular signaling domains, which would suggest that FGFR1 signaling may have a prominent role in tumorigenesis and FGF23 secretion, and thus be a therapeutic target in TIO.^(^ ^12^, ^13^ ^)^

Infigratinib (BGJ398; QED Therapeutics, San Francisco, CA, USA) is an orally bioavailable, FGFR1‐3 tyrosine kinase inhibitor (TKI) that has been studied in phase 2 trials of cancers with FGFR molecular alterations,^(^ ^14^, ^15^, ^16^, ^17^ ^)^ and was recently US Food and Drug Administration (FDA)‐approved for the treatment of previously treated cholangiocarcinoma with FGFR2‐gene fusions or rearrangements. In non‐TIO cancers, hyperphosphatemia is a common dose‐limiting side effect of infigratinib treatment due to blockade of FGFR1 and the associated inhibition of FGF23 signaling.^(^ ^18^ ^)^

We recently described the use of infigratinib in a patient with metastatic TIO. The patient experienced significant decreases in FGF23, normalization of blood phosphate levels, and a profound reduction in tumor burden while on therapy.^(^ ^19^ ^)^ Remarkably, pretreatment and posttreatment biopsies of PMT metastases showed evidence of infigratinib‐induced differentiation of the PMT into mature lamellar bone. Although the marked biochemical and structural responses reversed with cessation of therapy in this patient with diffuse metastatic disease, we hypothesized that in less aggressive forms of TIO, infigratinib may be either tumoricidal or induce the differentiation of benign PMTs to osseous tissue.

In this open‐label, phase 2, proof‐of‐principle trial, we treated a small cohort of patients with nonsurgical tumor‐induced osteomalacia with infigratinib for 24 weeks. The primary endpoint was the induction of biochemical remission, as indicated by persistent normalization of blood phosphate and FGF23 after discontinuation.

## Patients and Methods

This was an investigator‐sponsored, open‐label, single site, phase 2 trial (NCT03510455) approved by the Institutional Review Board of the National Cancer Institute, National Institutes of Health (NIH). It was conducted in accordance with the principles established by the Declaration of Helsinki. Written informed consent was obtained from participants. The study is registered with ClinicalTrials.gov (NCT03510455).

### Study participants

Adults with a clinical diagnosis of TIO due to nonlocalized, nonresectable, or metastatic tumor(s), or patients with resectable tumors that could not be removed by minor surgical procedures were eligible for participation. Patients were recruited from February 2019 to June 2019. Patients were excluded if they had genetic or secondary causes of hypophosphatemia, recent non‐TIO malignancy, current ophthalmologic disease, prior treatment with an FGFR inhibitor other than infigratinib, or use of interfering medications. Additional exclusion criteria included comorbid diseases that impair gastrointestinal absorption, prior history of corneal or retinal disorders, active cardiac or cerebrovascular disease, and insufficient bone marrow, hepatic, or renal function (creatinine >1.5× upper limit of normal [ULN] and/or estimated glomerular filtration rate [eGFR] <45 mL/min/1.73 m^2^). Pregnant or lactating women or patients under age 21 years with open growth plates were also ineligible.

### Infigratinib regimen

Infigratinib (BGJ398) was provided by QED Therapeutics. After a screening evaluation, subjects were admitted to the NIH Clinical Center. Calcitriol and phosphate supplements were discontinued 3 days prior to initiation of infigratinib. Phosphate and calcitriol were subsequently given only during dose interruptions, as needed. To prevent dry eyes and palmar‐plantar erythrodysesthesia, common TKI side effects, preservative‐free artificial tears (carboxymethylcellulose 0.5%), and ammonium lactate 12% lotion were provided to patients prophylactically.

Subjects received oral daily doses of infigratinib for up to 24 weeks. The initial dose of 75 mg daily was based on studies investigating infigratinib use in subjects with cholangiocarcinoma, as well as our clinical experience with two NIH patients with TIO who had previously participated in an infigratinib trial outside of the NIH (ClinicalTrials.gov NCT02160041). After 8 weeks on therapy, dose escalation and de‐escalation of drug was determined by investigators based on tolerability and changes in C‐terminal FGF23 (cFGF23) levels. Planned dose escalation would occur by 25 mg every 8 weeks to a maximum dose of 125 mg/d if cFGF23 levels were not suppressed to <100 relative units (RU)/mL. De‐escalations or interruptions would occur if a subject experienced an adverse event, including hyperphosphatemia. A cFGF23 assay that measures both the active intact FGF23 molecule and inactivated C‐terminal fragments was used for titration purposes rather than an intact FGF23 (iFGF23) assay because the cFGF23 assay was the only commercially available option at the time.

Because infigratinib routinely causes hyperphosphatemia in non‐TIO cancer treatment, oncologic treatment protocols use 21‐day cycles of 3 weeks on and 1 week off therapy to avoid prolonged hyperphosphatemia. However, because patients with TIO are much less likely to experience dose‐limiting hyperphosphatemia, in this study continuous dosing was used to maximize treatment efficacy.

### Biochemical assessments

Serial sampling was performed every 2 hours for 24 hours after the first dose of infigratinib to evaluate the pharmacokinetics and pharmacodynamics of the drug. Subsequently, laboratory tests were performed every 2 weeks. Routine blood and urine chemistries were performed at the NIH Clinical Center Department of Laboratory Medicine during inpatient stays and outpatient visits. Laboratory testing in the intervening weeks were performed either at the NIH or at a local laboratory, depending on the patient's proximity to the NIH. Infigratinib levels were also measured at weeks 4 and 8.

Biochemical testing included measurement of routine chemistries, 1,25D (radioimmunoassay; Mayo Medical Laboratories, Rochester, MN, USA), cFGF23 (ELISA; Immutopics International, San Clemente, CA, USA; Mayo Medical Laboratories), iFGF23 (ELISA; Immutopics International), C‐telopeptide (CTx) (electrochemiluminescence immunoassay; Mayo Medical Laboratories), and procollagen 1 intact N‐terminal (P1NP) (competitive radioimmunoassay; Mayo Medical Laboratories). Infigratinib levels were measured by Q2 Lab Solutions (Durham, NC, USA).

Tubular reabsorption of phosphate (TRP) was calculated using the following equation: 100 × [1 – (urine phosphate × serum creatinine)/(serum phosphate × urine creatinine)] and maximum renal tubular phosphate reabsorption in mass per unit volume of glomerular filtrate (TmP/GFR) was determined using the following equations: for TRP ≤0.86 (86%): TmP∕GFR = TRP × phosphate; for TRP >0.86 (86%): TmP∕GFR = (0.3 × TRP)/[1 − (0.8 × TRP)] ×phosphate.^(^ ^20^ ^)^

At the end of week 24, patients still on therapy were admitted again to the NIH Clinical Center to monitor off‐drug effects with daily blood and urine sampling × 7 days. Subjects with persistent biochemical remission after 7 days would be monitored as outpatients off therapy for 12 additional weeks. Subjects with normal phosphate levels on infigratinib but without persistent biochemical response on cessation were considered for an optional extension phase.

### Imaging

Renal ultrasounds were performed at baseline and at 24 weeks, and echocardiograms were performed at baseline, 8 weeks, and 24 weeks. Electrocardiograms (EKGs) were performed at screening and every 4–12 weeks depending on risk factors for arrhythmia.

In patients with identifiable tumors, recent prior imaging was obtained from the patient record. The imaging studies that best characterized their tumor prior to therapy (18Flouride‐Fluorodeoxyglucose (FDG) DG‐PET/CT and/or 68Gallium (68Ga)‐DOTATATE‐PET/CT) were repeated at the 24‐week visit, if clinically indicated, and the changes were assessed.

### Physical and quality of life assessments

Safety, tolerability, and efficacy were evaluated during in‐person visits every 4 weeks at the NIH Clinical Center and included a physical exam, ophthalmologic exam, patient‐reported outcome measures and strength assessments. The Disabilities of Arm Shoulder and Hand Outcome Measurement (DASH) questionnaire was used to measure self‐reported upper extremity symptoms and disability. The RAND 36‐item Short Form Health Survey (RAND SF‐36) version 1.0 was performed to determine effects of infigratinib on health‐related quality of life over the duration of the study. Patient Reported Outcomes Measurement Information System (PROMIS) item banks for fatigue (PROMIS SF v1.0‐Fatigue 8a), mobility (PROMIS Bank v2.0 – Mobility), and pain interference (PROMIS SF v1.1‐Pain Interference 8a) were also performed. Strength assessments included a Six‐Minute Walk Test, Grip Strength assessment, Pinch Strength assessment, and Five Time Sit to Stand. The Grip Strength assessment was performed on the dominant hand using a JAMAR Hand Dynamometer (Sammons Preston, Inc., Bolingbrook, IL, USA) and three successive trials were averaged to determine the final score. The Pinch Strength assessment was performed using the Exacta Hydraulic Pinch Gauge (North Coast Medical, Inc., Morgan Hill, CA, USA) with three successive trials averaged for the final score.

Patients were monitored via telephone between NIH visits.

### Statistical analyses

The primary objective of the study was to induce a persistent metabolic remission, defined by normalization of FGF23 and phosphate after study drug discontinuation. Secondary objectives included evaluating the effects of infigratinib on mineral homeostasis, radiographic appearance of PMTs, quality of life, and muscle strength. Key biochemical end points included changes in iFGF23, cFGF23, phosphate, 1,25D, TRP, and TmP/GFR. Because the study closed prematurely with only four patients enrolled, results were primarily descriptive. Adverse events were summarized by frequency and percentage. Continuous variables were presented as mean ± standard deviation (SD). Linear mixed effects models were fitted to the repeated measures data collected from the 24‐hour serial sampling and the first 8 weeks of therapy when the patients were on the same infigratinib dose (75 mg daily) because, after this point, dose reductions and interruptions occurred. Analysis was performed using SAS (version 9.4; SAS Institute, Inc., Cary, NC, USA). Graphs were produced using GraphPad Prism 8 (GraphPad Software, Inc., La Jolla, CA, USA).

## Results

Four subjects were screened, and all were eligible. Three subjects completed the 24‐week on‐drug period. Of these, subjects 1 and 2 experienced intermittent dose interruptions due to AEs. Subject 1 experienced three dose interruptions for a total of 25 days off therapy, and subject 2 experienced one 16‐day dose interruption. Subject 3 stopped the trial early after 17 weeks on drug due to AEs and was not restarted due to a persistently elevated cFGF23 on therapy. Subject 4 was on drug without interruption for 24‐weeks. After she was discontinued, she was restarted in the extension phase and remained on drug for 44 additional days but stopped due to failure to follow up.

### Baseline characteristics

Baseline demographics and biochemical findings are shown in Table 1. The average age was 34 years (range, 27–41 years). Subject 1 had a tumor in the C7 vertebra that persisted in spite of a previous surgery. Molecular testing on the pathologic tissue was attempted, but DNA/RNA quality was inadequate. Subject 2 had a tumor at the greater trochanter of the femur and did not wish to pursue surgery due to concerns over postsurgical morbidity. Because Subjects 3 and 4 were younger and had nonlocalized disease, genetic evaluations had been performed and were negative for the most common genetic causes of FGF23 excess including pathogenic variants in *PHEX* and *FGF23*. Patients had the expected biochemical findings of FGF23 excess: elevated intact and C‐terminal FGF23, hypophosphatemia, and low or inappropriately normal 1,25D. All but subject 3 had a baseline TRP lower than the normal range of 85%–95%; however, it was still inappropriately normal for her level of hypophosphatemia. No patients had hyperparathyroidism at the time of enrollment. Baseline CTx was slightly elevated in subject 2 but otherwise normal in other subjects. P1NP was greater than twice the upper limit of normal for subject 3 but was normal in other subjects. Alkaline phosphatase was elevated in subjects 3 and 4, but normal in subjects 1 and 2.

**Table 1:** Baseline Characteristics and Biochemistry

Subject	Age	Sex	Race	Tumor location	TIO duration	FGF23		Phosphate	1,25 (OH)_2_D	TRP	P1NP (μg/L)	C‐telopeptide (pg/mL)	Alkaline phosphatase
						Intact	C‐terminal						
Normal range/units	years				years	<52 pg/mL	<180 RU/mL	2.5–4.5 mg/dL	20–79 pmol/L	85%–95%	μ/L^a^	pg/mL^a^	35–105 U/L
1	41	M	W	C7 vertebra	10	1459	500	1.2	6	61	35	261	87
2	40	M	B	Greater trochanter of the femur	6	3415	2170	1.5	12	42	67	786	89
3	27	F	B	Nonlocalized	10	105	216	1.7	32	92	187	425	191
4	27	F	W	Nonlocalized	10	556	370	1.8	40	72	62	414	161

All subjects discontinued calcitriol and phosphate 3 days prior to baseline labs.

1,25(OH)_2_D = 1,25(OH)_2_ vitamin D; 25(OH)D = 25‐OH vitamin D; B = black; FGF23 = fibroblast growth factor 23; iPTH = intact parathyroid hormone; P1NP = propeptide of type 1 procollagen; TmP/GFR = ratio of the maximum rate of tubular phosphate reabsorption to the glomerular filtration rate; TRP = tubular reabsorption of phosphate; W = white.

^a^ Gender and age‐specific normal ranges: C‐telopeptide male 31–50 years: 93–630 pg/mL; C‐telopeptide female premenopausal: 25–573 pg/mL; P1NP male: 22–87 μ/L; P1NP female: 19–83 μ/L.

### Biochemical response

Overall, no subject achieved the primary objective of the study, enduring metabolic remission, defined as persistently normal FGF23 and blood phosphate following drug discontinuation. Subjects 1, 3, and 4 experienced a complete metabolic response while on therapy, although this reversed rapidly with drug cessation. Subject 2 achieved a significant biochemical response; however, neither his intact nor C‐terminal FGF23 ever normalized.

**Fig. 1:**
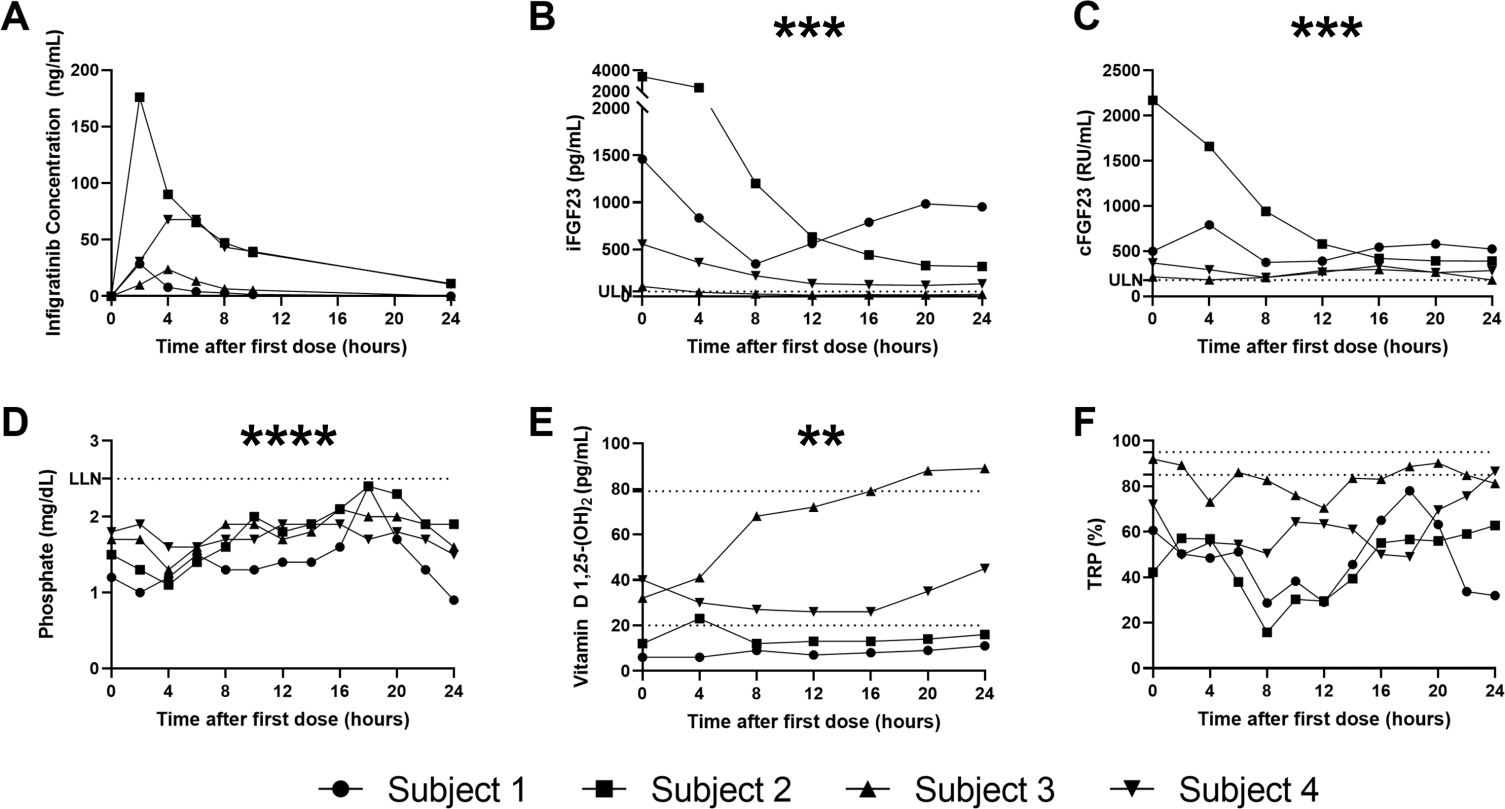
Twenty‐four‐hour pharmacokinetics and pharmacodynamics following the initial infigratinib 75 mg dose. (*A*) Infigratinib concentration, (*B*) iFGF23, (*C*) cFGF23, (*D*) phosphate, (*E*) 1,25D, (*F*) TRP. Horizontal dotted lines indicate the ULN and LLN. iFGF23, cFGF23, phosphate, and 1,25D showed a significant change from baseline (0 hour) using linear mixed effects modeling. *p < 0.05; **p < 0.01; ***p < 0.001; ****p < 0.0001. LLN = lower limit of normal; ULN = upper limit of normal.

The intrasubject 24‐hour pharmacokinetic response to the first dose of infigratinib (75 mg) was highly variable (Fig. 1*A*). Subjects 2 and 4 had high blood levels of infigratinib with longer half‐lives; subjects 1 and 3 had relatively lower blood levels of infigratinib with shorter half‐lives (Table 2). During the first 24 hours of infigratinib, iFGF23 decreased significantly (*p* < 0.001, Fig. 1*B*). Subject 3, who had the lowest baseline iFGF23, experienced reduction of iFGF23 into the normal range, despite a relatively low blood concentration of infigratinib. Subject 1, who also had relatively lower levels of blood infigratinib, experienced an initial decrease in iFGF23, which started to rise again after 8 hours, when infigratinib levels were very low. In contrast to the decrease in iFGF23, cFGF23 levels, which reflect both active intact FGF23 and inactive C‐terminal fragments, were unchanged or slightly increased in three subjects, with the notable exception of subject 2 who had an 82% reduction in cFGF23 (Fig. 1*C*), paralleling the similarly profound drop in iFGF23. Phosphate levels increased on infigratinib (Fig. 1*D*, *p* < 0.0001), but no phosphate exceeded the lower limit of normal at hour 24. 1,25D was minimally changed in all patients except for subject 3, who had an increase in 1,25D above the upper limit of normal (Fig. 1*E*). TRP response was variable without a clear trend over the first 24 hours of therapy (Fig. 1*F*).

**Table 2:** Pharmacokinetics Following a Single Oral Dose of Infigratinib 75 mg

Variable	Subject 1	Subject 2	Subject 3	Subject 4
Pharmacokinetics				
T_1/2_ (hour)	2.73	7.75	3.02	7.62
T_max_ (hour)	2	2	4	6
C_max_ (ng/mL)	28.6	176	23.8	67.9
24 hour AUC (hour*ng/mL)	89.68	1094.1	133.15	764.02

AUC = area under the curve; C_max_ = peak infigratinib concentration; T_1/2_ = infigratinib half‐life; T_max_ = time to maximum infigratinib concentration.

**Fig. 2:**
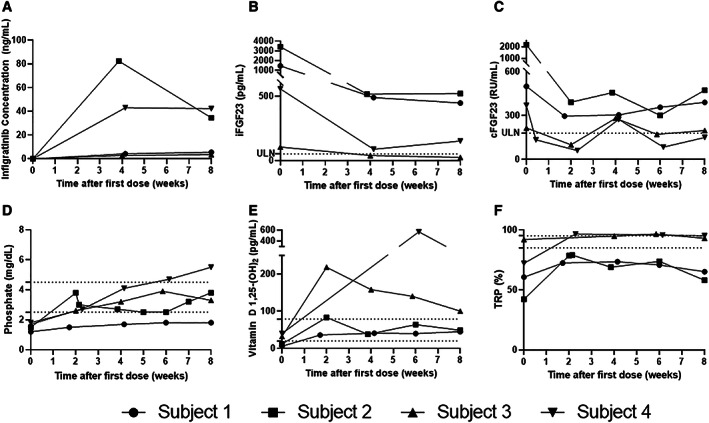
Pharmacokinetics and pharmacodynamics over the first 8 weeks on fixed dosing of infigratinib 75 mg daily. (*A*) Infigratinib concentration, (*B*) iFGF23, (*C*) cFGF23, (*D*) phosphate, (*E*) 1,25D, (*F*) TRP. Horizontal dotted lines indicate the ULN and LLN. Linear mixed effects modeling demonstrated significant changes from baseline in cFGF23, phosphate, and 1,25D. *p < 0.05; **p < 0.01; ***p < 0.001; ****p < 0.0001. LLN = lower limit of normal; ULN = upper limit of normal.

For the first 8 weeks of therapy, subjects were treated with infigratinib 75 mg daily (Fig. 2*A–F*) followed by individualized titration. Consistent with the pharmacokinetic findings over the first 24 hours, at weeks 4 and 8, infigratinib levels were much higher in subjects 2 and 4. Predictably, dose reductions were necessary at week 8 due to TKI‐related side effects in subject 2 and hyperphosphatemia in subject 4, whereas the two subjects with lower infigratinib levels required dose escalation. Nevertheless, all patients achieved a partial biochemical response on 75 mg daily at week 8. iFGF23 decreased in all patients but entered the normal range in subject 3 only. Subjects 3 and 4 had cFGF23 levels normalize during the first 8‐week period. As expected, phosphate increased, with phosphate levels in or above the normal range at 8 weeks. 1,25D levels were above the normal range in the two subjects who had normalization of cFGF23, and in the normal range for the two other subjects. TRP increased in all patients by week 8. Bone turnover markers were variable throughout the treatment period, with relative increases in CTx, P1NP, and alkaline phosphatase observed in several subjects (Fig. S1).

During the rest of the trial, patient dosages and courses diverged due to adverse events with resultant dose interruptions and adjustments. Maintenance infigratinib doses at 24 weeks were as follows: subject 1, 75 mg daily; subject 2, 50 mg daily; subject 4, 25 mg daily. Subject 3 was on 50 mg daily at 17 weeks when infigratinib was discontinued. Only subject 4 continued into the extension phase for an additional 44 days. Detailed descriptions of each patient's course are included in the online Supporting Information (Figs. S2, S3, S4, S5).

### Radiographic response

**Fig. 3:**
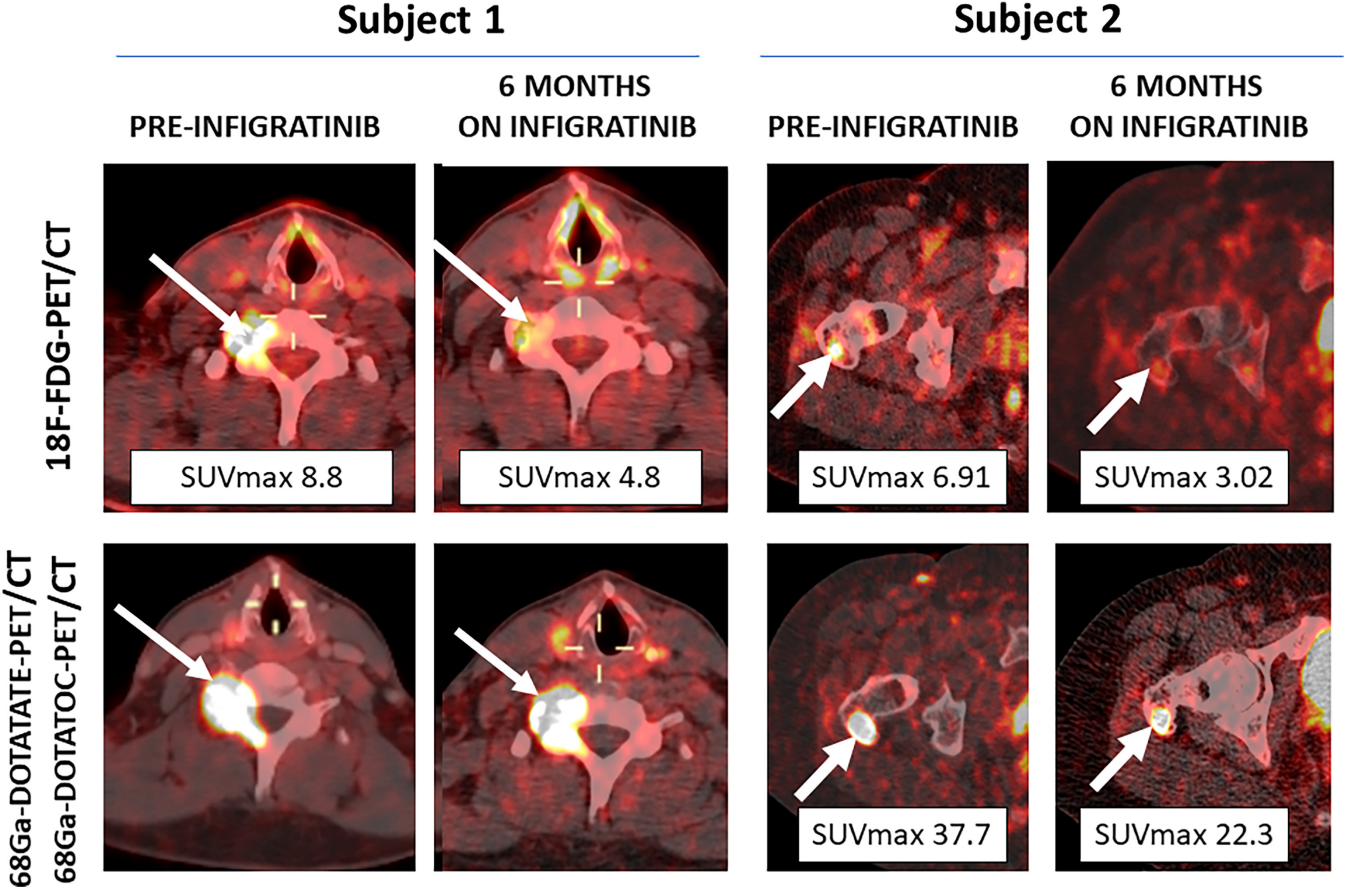
18F‐FDG‐PET/CT, 68Ga‐DOTATATE‐PET/CT, and 68Ga‐DOTATOC‐PET/CT scans from subjects 1 and 2 prior to infigratinib initiation and after 6 months of therapy. Phosphaturic mesenchymal tumors of the C7 vertebra (subject 1) and right greater trochanter (subject 2) are indicated by white arrows. Subject 1 had a pre‐infigratinib 68Ga‐DOTATOC‐PET/CT and post‐infigratinib 68Ga‐DOTATATE‐PET/CT, limiting quantitative comparison of the lesions, although there was a subjective decrease in uptake. In all other studies, the SUVmax of the PMTs were reduced by greater than 40% after treatment, without appreciable change in size. SUVmax = maximum standardized uptake value.

Patients had 68Ga‐DOTATATE‐PET/CT and 18F‐FDG‐PET/CT scans in the week following medication cessation. The previously identified tumors in subjects 1 and 2 had decreased relative uptake on both imaging modalities, when compared to pretreatment imaging (Fig. 3). Causative tumors continued to be nonlocalized in subjects 3 and 4.

### Physical and quality‐of‐life assessments

Strength and patient‐reported outcomes were limited by the small number of subjects, and intermittent dose interruptions that occurred starting at 8 weeks, precluding the utility of statistical analyses. Nevertheless, there was a suggestion of small improvements in the four strength assessments at 24 weeks (Table S1).

At the 8‐week time point, when patients had been on infigratinib 75 mg daily for 8 weeks, quality‐of‐life assessments appeared to improve, including a reduction in upper extremity disability using the DASH questionnaire, in the eight quality‐of‐life domains using the RAND SF‐36, and decreased fatigue, decreased pain interference, and improved mobility using PROMIS (Table S1).

By 24 weeks, patients had experienced dose reductions and subject 3 was no longer on infigratinib. At this time point, quality‐of‐life assessments appeared to be slightly worse compared to 8 weeks, and some were worse than baseline, including RAND SF‐36 quality‐of‐life domains: physical functioning, role limitations due to physical and emotional health, emotional well‐being, energy and fatigue, and bodily pain. There was wide variation in these quality‐of‐life assessments over time, which likely reflects small sample size, dose interruptions, and intermittent adverse events. Of note, on week 20, the RAND SF‐36 assessments were better than baseline across all domains.

### Safety and study cessation

Subjects experienced mild to moderate treatment‐related adverse events on infigratinib. No subjects experienced serious adverse events. There were 111 AEs, with 87 considered possibly, probably, or definitely related to infigratinib. Most AEs were Common Terminology Criteria for Adverse Events (CTCAE) grade 1–2; the only grade 3 AEs were increased alanine aminotransferase and decreased neutrophil count in subject 2, both of which improved following drug interruption. A comprehensive table of adverse events is included in Table S2.

Ocular AEs were common and the eye findings from this study were recently reported.^(^ ^21^ ^)^ To summarize, all patients developed corneal keratitis with blurred vision; drug interruptions were required in two patients. Vision normalized within 60 days of infigratinib discontinuation; however, two patients had residual corneal scarring, localized outside of the visual axis. Although vision was completely restored in these patients, corneal scarring has the potential to cause permanent vision loss. Because of the higher‐than‐expected rate and severity of corneal keratitis, and the lack of meeting the primary endpoint (permanent remission after 6 months of therapy), recruitment was halted and infigratinib therapy of the one subject in the extension phase was discontinued.

Other adverse events related to the drug were hyperphosphatemia, typical TKI side effects, or those determined to be unrelated to the study drug. TKI‐related adverse events included blurred vision, nail changes, dry eyes, dry mouth, weight loss, mucositis, dysgeusia, hair changes, trichomegaly, and decreased blood cell counts.

## Discussion

Infigratinib resulted in a transient improvement of biochemical markers in four subjects with TIO; however, this response entirely reversed after drug cessation and no subjects achieved persistent biochemical remission. There was a mild reduction in radionuclide uptake on functional imaging but no change in tumor size in the patients with identifiable tumors. Strength assessments and patient‐reported outcomes were suggestive of small improvements at 8 weeks, that reversed by week 24. AEs were mild to moderate, with no serious events; however, ocular side effects were found in all subjects. The study ultimately closed early due to a greater‐than‐expected incidence of ocular keratitis, likely due to continuous dosing, and evidence that permanent remission was not achieved in the initial four subjects.

The identification of fusion genes in PMT surgical specimens was a pivotal step in understanding PMT pathogenesis. FN1‐FGFR1, the most common translocation, found in 40% of PMTs, first identified FGFR1 blockade as a promising potential therapeutic target for TIO. An additional 6% of PMTs harbor a FN1‐FGF1 translocation, which includes fibroblast growth factor 1, a ligand of FGFR1 and other FGF receptors. Although this fusion is unique, it likely results in increased activation of the FGFR1 pathway, similar to the FN1‐FGFR1 fusion.^(^ ^12^ ^)^ Recently, fusion‐negative PMTs have been found to have increased expressions of *KL* mRNA, which encodes alpha‐KLOTHO, the coreceptor for FGF23‐FGFR1 binding, hinting at a third possible mechanism for PMT tumorigenesis, upstream of the FGFR1 pathway.^(^ ^22^, ^23^ ^)^ All three mechanisms presumptively involve increased activation of FGFR1, causing PMT tumorigenesis and FGF23 excess. For this reason, it is plausible that TIO tumors may respond to FGFR1 blockade with certain TKIs. Of note, cases of nonlocalizable TIO do not have available pathologic material and the pathogenesis of this subset of TIO is still unknown. However, in this study, the two cases of nonlocalized PMTs also responded effectively to FGFR1 blockade, revealing that FGFR1 is indeed important in this subset of patients as well. The patients with localized PMTs did not have available pathologic tissue for fusion testing, but their robust biochemical response to the drug confirmed the importance of FGFR1 in their tumors.

Our previous experience treating a patient with diffusely metastatic TIO with a known FN1‐FGFR1 fusion with infigratinib resulted in FGF23 suppression and normalization of phosphate, TRP, and 1,25D. Of note, a tumor adjacent to the scapula calcified during the first 3 months of infigratinib treatment. Pretreatment and posttreatment biopsies showed an apparent metaplastic redifferentiation from a sarcomatous‐appearing PMT to mature lamellar bone.^(^ ^19^ ^)^ This indicates that PMTs are likely derived from skeletal stem cells and that FGFR1 may hold a pivotal role in tumor differentiation.

In the current study, subjects developed ocular keratitis while on infigratinib, with corneal scarring occurring in 50%.^(^ ^19^ ^)^ In studies of subjects with cholangiocarcinoma, infigratinib causes dry eye, including keratitis, in 44% of patients, and blurred vision in 21%, with no reactions above grade 2.^(^ ^24^ ^)^ The increased frequency and severity of the effect of infigratinib on the cornea in our study may be due to continuous dosing, which was unique to this protocol.^(^ ^21^ ^)^ The risk–benefit assessment in patients with benign TIO who are comparatively healthy precludes long‐term continuous dosing of infigratinib due to the potential of inducing permanent eye damage. In cases of TIO where the goal is not curative due to advanced metastatic disease, cyclical dosing might be preferable, as this may reduce the risk of ocular keratitis and scarring. Autologous serum eye drops, produced prior to initiation of FGFR1 blockade, may also be helpful in replenishing growth factors in the eye and avert keratitis; however, the efficacy of this therapy is still under investigation.^(^ ^21^ ^)^

This study was limited by the small sample size due to the rarity of the underlying disease and early closure. Furthermore, adverse effects necessitated dose reductions or interruptions, limiting optimal drug exposure. Intersubject variability in pharmacokinetics as well as differing baseline disease severity likely contributed to a wide range of dose requirements and responses. Baseline PET scans were not standardized and, in one case, was performed at an outside facility limiting the ability to compare pretherapy and posttherapy imaging. The interpretations of quality‐of‐life outcomes and strength data were limited by the small sample size and high variability due to transient drug‐related AEs experienced by patients during the study.

FGFR1 blockade with infigratinib effectively ameliorated the biochemical findings in TIO, decreasing tumoral FGF23 production and normalizing mineral metabolism. The biochemical response observed in this study confirms the role of FGFR1 signaling in PMT pathogenesis, including in cases of nonlocalized TIO for which the molecular drivers could not be assessed. However, infigratinib's effect reverses after drug cessation, suggesting that the drug is not tumoricidal with the regimen implemented. Although it is possible that a longer course of therapy at higher doses could induce a persistent remission, this approach would likely be limited by untoward treatment‐related AEs. Therefore, long‐term therapy should not be advocated given the side‐effect profile, except perhaps in cases of metastatic disease. In the future, more targeted therapeutics, such as monoclonal antibodies or a more specific TKI, that blocks FGFR1 with fewer off‐target effects, might allow for higher doses and longer continuous therapy in this population.

## Author Contributions

**Iris R. Hartley:** Conceptualization; data curation; formal analysis; investigation; methodology; writing – original draft; writing – review and editing. **Kelly L. Roszko:** Conceptualization; data curation; investigation; writing – review and editing. **Xiaobai Li:** Formal analysis; writing – review and editing. **Karen Pozo:** Data curation; project administration; writing – review and editing. **Jamie Streit:** Data curation; project administration; writing – review and editing. **Jaydira Del Rivero:** Conceptualization; data curation; investigation; writing – review and editing. **M. Teresa Magone:** Conceptualization; data curation; investigation; writing – review and editing. **Michaele R. Smith:** Conceptualization; data curation; writing ‐ reveiw and editing. **Roo Vold:** Writing – review and editing. **Carl L. Dambkowski:** Writing – review and editing. **Michael T. Collins:** Conceptualization; data curation; formal analysis; investigation; methodology; supervision; writing – review and editing.

## Conflict of Interest

NIDCR receives research funding from QED Therapeutics as a part of a Collaborative Research and Development Agreement (CRADA), RV and CD are employees of QED Therapeutics.

### Peer Review

The peer review history for this article is available at https://publons.com/publon/10.1002/jbm4.10661.

## Supporting information


**Fig. S1.** Markers of bone turnover over 24 weeks. (*A*) Alkaline phosphatase. (*B*) CTx. (*C*) P1NP. Individual lab values are represented by black symbols, while gray boxes represent the infigratinib dose averaged over the 4 patients. Male and female upper limit of normal is represented by horizontal dotted lines. Normal ranges were as follows: Alkaline phosphatase 35–105 U/L; CTx male 31–50 years: 93–630 pg/mL; CTx female premenopausal: 25–573 pg/mL; P1NP male: 22–87 mcg/L; P1NP female: 19–83 mcg/L.
**Figs. S2–S5:** Pharmacokinetics and pharmacodynamics of individual subjects over the course of the study including follow up. Measurements include (*A*) Infigratinib concentration, (*B*) iFGF23, (*C*) cFGF23, (*D*) phosphate, (*E*) 1,25D, and (*F*) TRP. Individual values are represented by black icons while gray boxes represent daily infigratinib dose. Reasons for dose reductions and dose interruptions are noted (*A*). Normal ranges are represented by horizontal dotted lines.
**Figure S2**: Subject 1 experienced relatively low absorption of infigratinib. He experienced normalization of iFGF23 and cFGF23 during weeks 18–20 after the dose was increased to 100 mg daily, however, he subsequently developed hyperphosphatemia, which prevented him from being maintained on this dose. After adjusting his dose down to 75 mg daily, his phosphate remained elevated until he was discontinued at week 24. On cessation of therapy, cFGF23 rebounded above baseline, and subsequent measurements iFGF23 and cFGF23 returned to baseline.
**Figure S3**: Subject 2 had the highest blood levels of infigratinib on 75 mg daily dose. His dose was limited by high ALT and low ANC on infigratinib 75 mg per day requiring dose cessation. He was then maintained on infigratinib 50 mg daily until 24 weeks. Although his iFGF23 and cFGF23 decreased 95% and 89% respectively from his baseline, both remained above the normal range with an iFGF23 nadir of 171 RU/mL (normal <54 pg/mL) and cFGF23 nadir of 241 RU/mL (normal <180 RU/mL). In response, blood phosphate increased only to the low‐normal range, and vitamin D 1,25 generally remained in the normal range. Although TRP increased, it never normalized.
**Figure S4**: Subject 3 responded within 24 hours to infigratinib, with normalization of iFGF23, phosphate elevation, and vitamin D 1,25 elevation even with very low blood infigratinib levels. However, cFGF23, although initially decreased, subsequently remained at or above the normal range. Since cFGF23 was used for dose adjustments, when subject 3 developed blurred vision requiring a dose interruption, the persistently high cFGF23 made it appear that the patient's tumor was not adequately responding to therapy. Elevated phosphate and 1,25D was attributed to the blockade of non‐tumoral FGFR1 resulting in a FGF23 resistance. After a discussion of risks and benefits, the investigators and patient decided not to resume infigratinib therapy.
**Figure S5**: Subject 4 developed hyperphosphatemia at 8 weeks requiring dose reduction to a final dose of 25 mg of infigratinib per day. On this dose, phosphate remained in the normal range without need for supplementation and 1,25D remained at or above the normal range. TRP increased initially to the normal range but subsequently decreased on lower doses. Although cFGF23 and iFGF23 dropped substantially initially, and cFGF23 normalized, on the lower infigratinib dose, both increased the rest of the treatment course. On cessation, phosphate and 1,25D decreased and iFGF23 increased to baseline. The patient enrolled in the extension period, however, was ultimately taken off the protocol after an additional 44 days on therapy due to loss to follow up.

**Table S1.** Patient reported outcomes and strength assessments.
**Table S2.** Adverse Events.
