# Supplementary material for: Infigratinib Reduces Fibroblast Growth Factor 23 (FGF23) and Increases Blood Phosphate in Tumor‐Induced Osteomalacia
Source: JBMR Plus. 2022 Jul 22;6(8):e10661. doi: 10.1002/jbm4.10661 (PMC9382865; doi:10.1002/jbm4.10661)
Supplement: Supplementary file 1 — Fig. S1. Markers of bone turnover over 24 weeks. (A) Alkaline phosphatase. (B) CTx. (C) P1NP. Individual lab values are represented by black symbols, while gray boxes represent the infigratinib dose averaged over the 4 patients. Male and female upper limit of normal is represented by horizontal dotted lines. Normal ranges were as follows: Alkaline phosphatase 35–105 U/L; CTx male 31–50 years: 93–630 pg/mL; CTx female premenopausal: 25–573 pg/mL; P1NP male: 22–87 mcg/L; P1NP female: 19–83 mcg/L. Figs. S2–S5: Pharmacokinetics and pharmacodynamics of individual subjects over the course of the study including follow up. Measurements include (A) Infigratinib concentration, (B) iFGF23, (C) cFGF23, (D) phosphate, (E) 1,25D, and (F) TRP. Individual values are represented by black icons while gray boxes represent daily infigratinib dose. Reasons for dose reductions and dose interruptions are noted (A). Normal ranges are represented by horizontal dotted lines. Figure S2: Subject 1 experienced relatively low absorption of infigratinib. He experienced normalization of iFGF23 and cFGF23 during weeks 18–20 after the dose was increased to 100 mg daily, however, he subsequently developed hyperphosphatemia, which prevented him from being maintained on this dose. After adjusting his dose down to 75 mg daily, his phosphate remained elevated until he was discontinued at week 24. On cessation of therapy, cFGF23 rebounded above baseline, and subsequent measurements iFGF23 and cFGF23 returned to baseline. Figure S3: Subject 2 had the highest blood levels of infigratinib on 75 mg daily dose. His dose was limited by high ALT and low ANC on infigratinib 75 mg per day requiring dose cessation. He was then maintained on infigratinib 50 mg daily until 24 weeks. Although his iFGF23 and cFGF23 decreased 95% and 89% respectively from his baseline, both remained above the normal range with an iFGF23 nadir of 171 RU/mL (normal <54 pg/mL) and cFGF23 nadir of 241 RU/mL (normal <180 RU/mL). In response [file JBM4-6-e10661-s002.docx]

**Supplemental Figure 1**


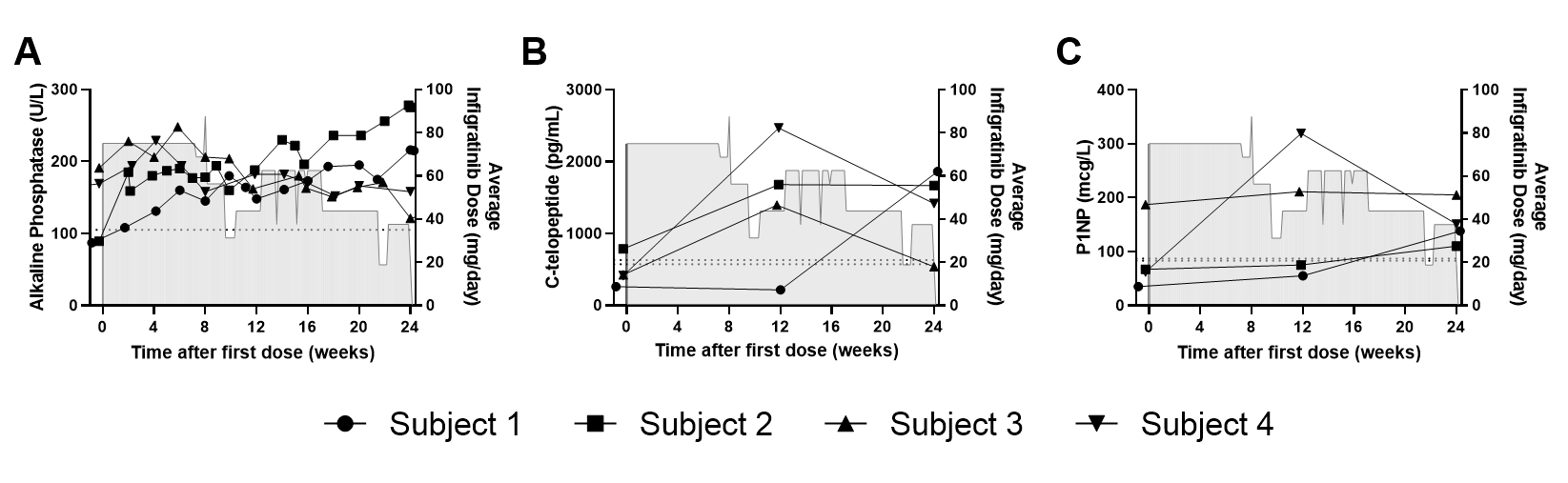


**Supplemental Figure 1**

Markers of bone turnover over 24 weeks. (A) Alkaline phosphatase. (B) Ctx. (C) P1NP. Individual lab values are represented by black symbols, while gray boxes represent the infigratinib dose averaged over the 4 patients. Male and female upper limit of normal is represented by horizontal dotted lines. Normal ranges were as follows: Alkaline phosphatase 35-105 U/L; Ctx male 31-50 years: 93-630 pg/mL; Ctx female premenopausal: 25-573 pg/mL; P1NP male: 22-87 mcg/L; P1NP female: 19-83 mcg/L**.**

**Supplemental Figures 2-5**

Pharmacokinetics and pharmacodynamics of individual subjects over the course of the study including follow up. Measurements include (A) Infigratinib concentration, (B) iFGF23, (C) cFGF23, (D) phosphate, (E) 1,25D, and (F) TRP. Individual values are represented by black icons while gray boxes represent daily infigratinib dose. Reasons for dose reductions and dose interruptions are noted (A). Normal ranges are represented by horizontal dotted lines.

**Supplemental Figure 2: Subject 1**


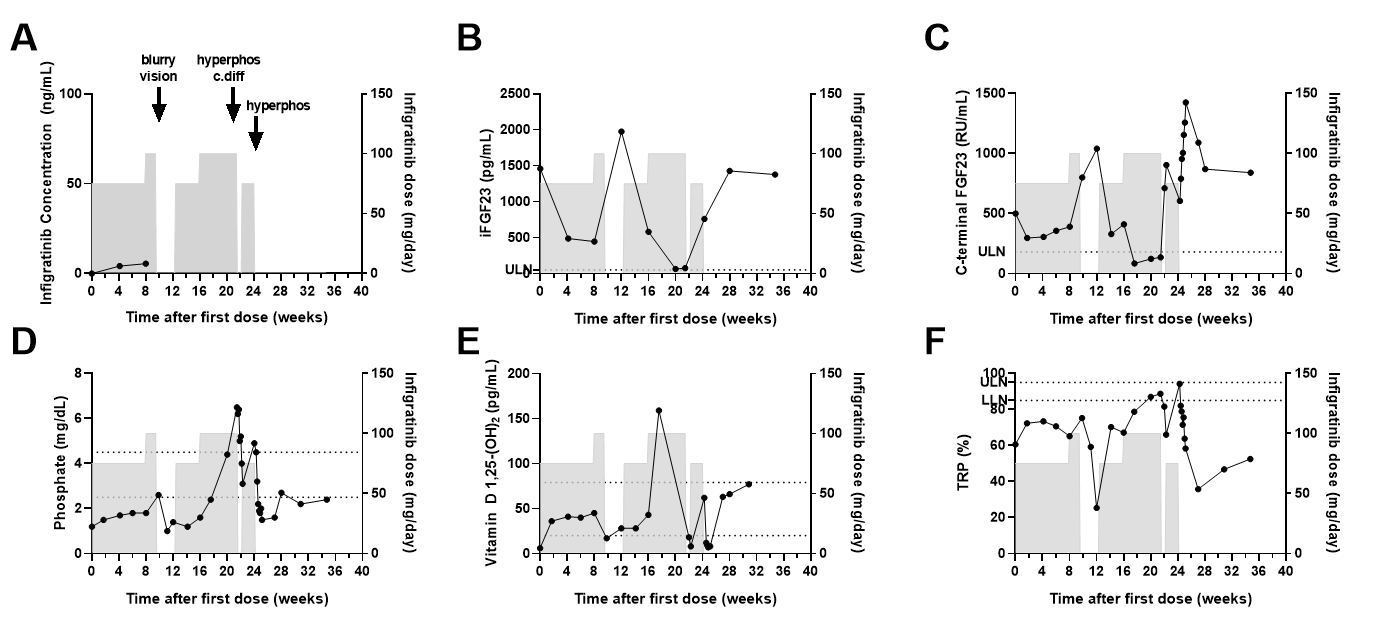


*Supplemental Figure 2:* Subject 1 experienced relatively low absorption of infigratinib. He experienced normalization of iFGF23 and cFGF23 during weeks 18-20 after the dose was increased to 100 mg daily, however, he subsequently developed hyperphosphatemia, which prevented him from being maintained on this dose. After adjusting his dose down to 75 mg daily, his phosphate remained elevated until he was discontinued at week 24. On cessation of therapy, cFGF23 rebounded above baseline, and subsequent measurements iFGF23 and cFGF23 returned to baseline.

**Supplemental Figure 3: Subject 2**


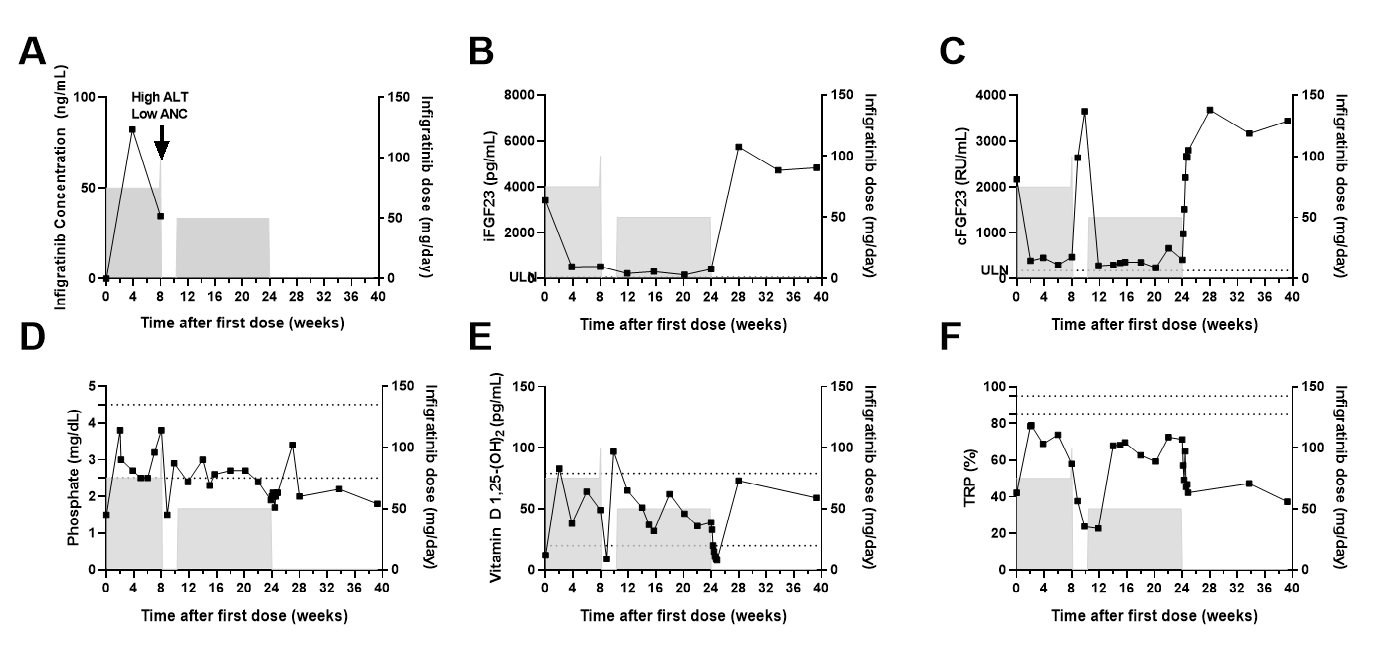


*Supplemental Figure 3:* Subject 2 had the highest blood levels of infigratinib on 75 mg daily dose. His dose was limited by high ALT and low ANC on infigratinib 75 mg per day requiring dose cessation. He was then maintained on infigratinib 50 mg daily until 24 weeks. Although his iFGF23 and cFGF23 decreased 95% and 89% respectively from his baseline, both remained above the normal range with an iFGF23 nadir of 171 RU/mL (normal <54 pg/mL) and cFGF23 nadir of 241 RU/mL (normal <180 RU/mL). In response, blood phosphate increased only to the low-normal range, and vitamin D 1,25 generally remained in the normal range. Although TRP increased, it never normalized.

**Supplemental Figure 4: Subject 3**


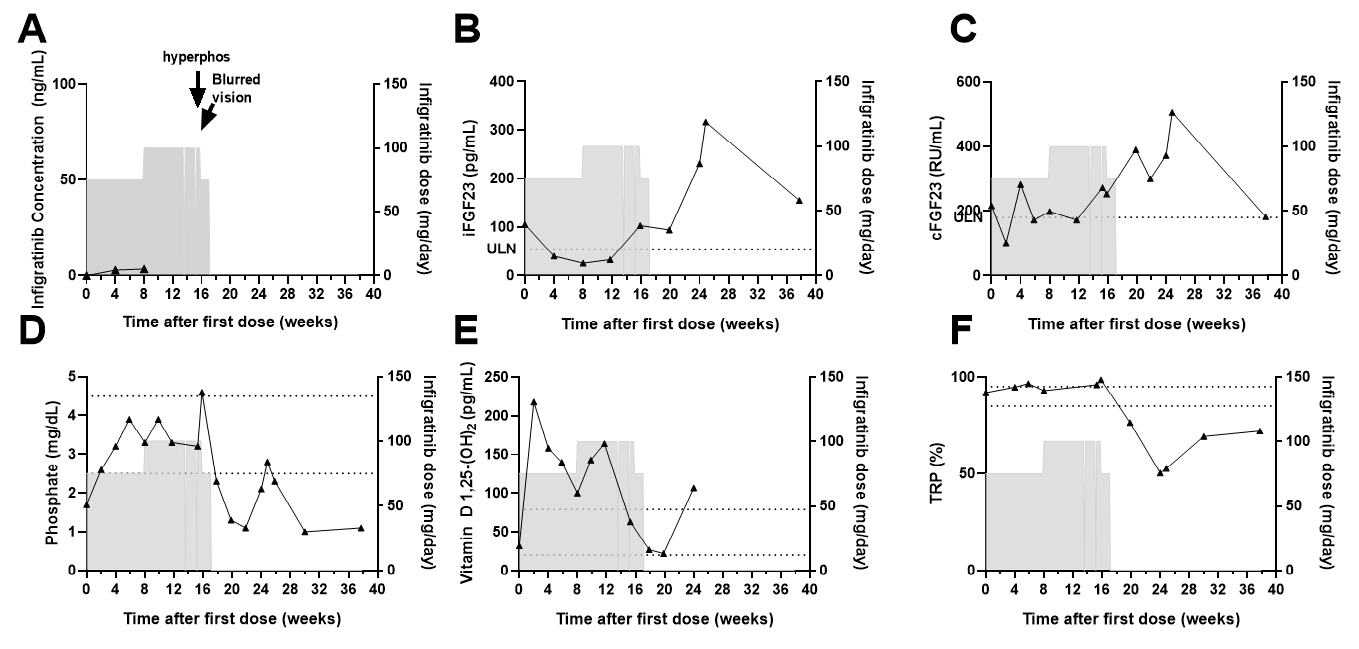


*Supplemental Figure 4:* Subject 3 responded within 24 hours to infigratinib, with normalization of iFGF23, phosphate elevation, and vitamin D 1,25 elevation even with very low blood infigratinib levels. However, cFGF23, although initially decreased, subsequently remained at or above the normal range. Since cFGF23 was used for dose adjustments, when subject 3 developed blurred vision requiring a dose interruption, the persistently high cFGF23 made it appear that the patient’s tumor was not adequately responding to therapy. Elevated phosphate and 1,25D was attributed to the blockade of non-tumoral FGFR1 resulting in a FGF23 resistance. After a discussion of risks and benefits, the investigators and patient decided not to resume infigratinib therapy.

**Supplemental Figure 5: Subject 4**


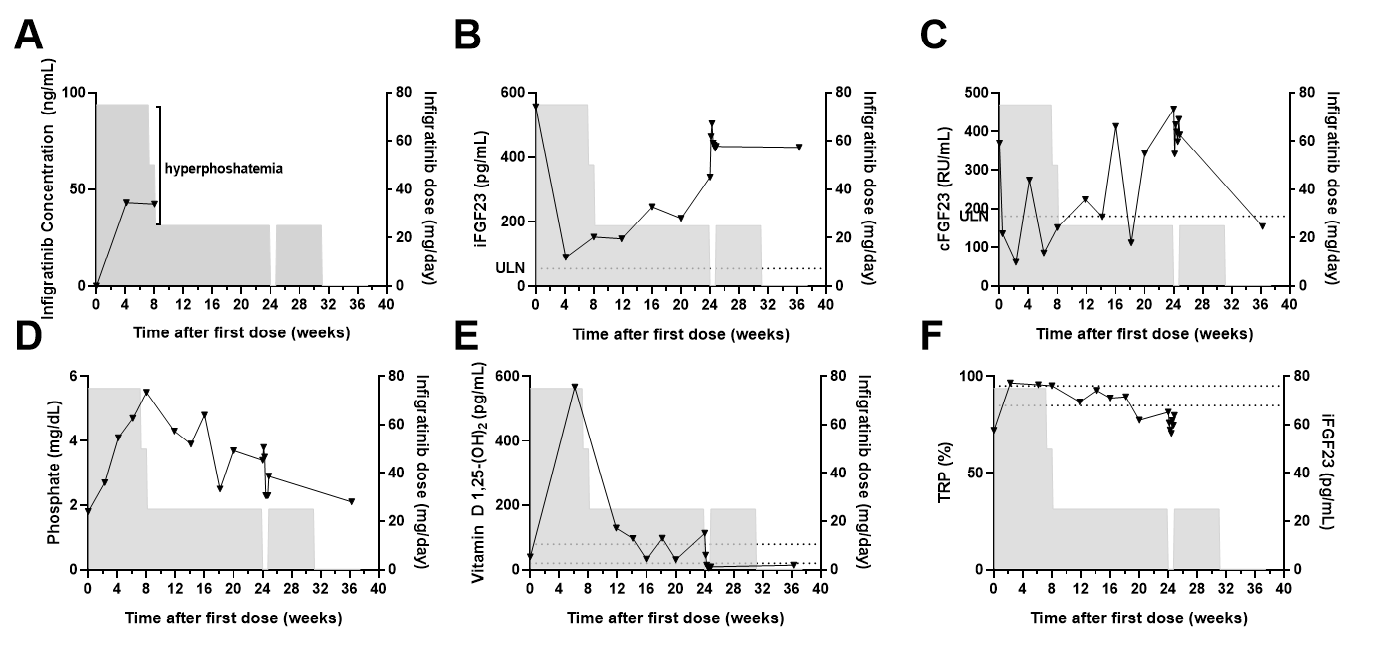


*Supplemental Figure 5:* Subject 4 developed hyperphosphatemia at 8 weeks requiring dose reduction to a final dose of 25 mg of infigratinib per day. On this dose, phosphate remained in the normal range without need for supplementation and 1,25D remained at or above the normal range. TRP increased initially to the normal range but subsequently decreased on lower doses. Although cFGF23 and iFGF23 dropped substantially initially, and cFGF23 normalized, on the lower infigratinib dose, both increased the rest of the treatment course. On cessation, phosphate and 1,25D decreased and iFGF23 increased to baseline. The patient enrolled in the extension period, however, was ultimately taken off the protocol after an additional 44 days on therapy due to loss to follow up.
