# Supplementary material for: Infigratinib Reduces Fibroblast Growth Factor 23 (FGF23) and Increases Blood Phosphate in Tumor‐Induced Osteomalacia
Source: JBMR Plus. 2022 Jul 22;6(8):e10661. doi: 10.1002/jbm4.10661 (PMC9382865; doi:10.1002/jbm4.10661)
Supplement: Supplementary file 2 — Table S1. Patient reported outcomes and strength assessments. Table S2. Adverse Events. [file JBM4-6-e10661-s001.docx]

Supplemental Table 1: Patient reported outcomes and strength assessments

| Patient Reported Outcomes | N=4 |
| --- | --- |
| Strength Assessments | |
| 6 Minute Walk Test (meters, Normal range 391-751 meters[1])  Baseline  24 Weeks | 474+/-250  496+/-173 |
| Grip Strength Evaluation (kilograms)  Baseline  8 Weeks  24 Weeks | 15+/-12  18+/-9  16+/-1 |
| Pinch Test (kilograms)  Baseline  8 Weeks  24 Weeks | 6.8 +/-4  6.8 +/-1  7+/-1 |
| 5x Sit to stand (Seconds)  Baseline  8 Weeks  24 Weeks | 15+/-7  14+/-1  11+/-1 |
| Quality of Life Assessments | |
| DASH  *Minimum score of 0 (no disability); Maximum of 100 (severe disability)*  Baseline  8 Weeks  24 Weeks | 16+/-5  14+/-5  12+/-8 |
| RAND SF-36  *Minimum score of 0; Maximum of 100; Higher score indicates a better outcome*  Physical Functioning  Baseline  8 Weeks  24 Weeks  RAND – Role limitations due to physical health problems  Baseline  8 Weeks  24 Weeks  RAND – Role limitations due to emotional health problems  Baseline  8 Weeks  24 Weeks  RAND – Emotional well-being  Baseline  8 Weeks  24 Weeks  RAND – Energy/Fatigue  Baseline  8 Weeks  24 Weeks  RAND – Social functioning  Baseline  8 Weeks  24 Weeks  RAND – Bodily Pain  Baseline  8 Weeks  24 Weeks  RAND – General health perceptions  Baseline  8 Weeks  24 Weeks | 46+/-19  51+/-17  40+/-6  44+/-13  68+/-31  25+/-50  67 +/-27  75+/-32  41.7+/-50  67+/-15  72+/-13  62+/-10  50+/-29  53+/-22  49+/-33  59+/-37  78+/-21  72+/-33  52+/-29  63+/-37  36+/-2.5  60+/-11  73+/-17  71+/-18 |
| PROMIS – Fatigue 8A Short Form  *T scores where mean is 50 and 1SD is 10, Higher score indicate increased fatigue*  Baseline  8 Weeks  24 Weeks | 55+/-8  53+/-8  52+/-9 |
| PROMIS – Pain Interference 8A short form  *T scores where mean is 50 and 1SD is 10, Higher score indicate increased pain interference*  Baseline  8 Weeks  24 Weeks | 56+/-4  52+/-6  53+/-9 |
| PROMIS – Mobility Item Bank  *T scores where mean is 50 and 1SD is 10, Higher score indicate a better outcome (unencumbered mobility)*  Baseline  8 Weeks  24 Weeks | 38+/-7  39+/-5  39+/-3 |

Supplemental Table 2: Adverse Events

| Total Number of Subjects |  | 4 |
| --- | --- | --- |
| Total Number of Adverse Events |  | 111 |
| System Organ Class  Preferred term | Relationship to drug | n (%) [AEs] |
| Total Subjects with any AE |  | 4 (100%) [111] |
|  |  |  |
| Blood and lymphatic disorders |  |  |
| Anemia | Possible | 1 (25%) [1] |
| Ear and labyrinth disorders |  |  |
| Ear pain | Unrelated | 1 (25%) [1] |
| Hearing impairment | Unrelated | 1 (25%) [1] |
| Eye disorders |  |  |
| Blurred vision | Possible | 4 (100%) [4] |
| Dry eye | Possible | 3 (100%) [3] |
| Corneal inflammation/keratitis | Probable/Definite | 4 (100%) [4] |
| Nuclear sclerosis | Possible | 1 (25%) [1] |
| Glaucoma | Unlikely | 1 (25%) [1] |
| Hypertrichosis of eyelashes | Probable | 2 (50%) [2] |
| Infections and Infestations |  |  |
| Rash pustular | Possible/Unrelated | 2 (50%) [2] |
| C. difficile colitis | Unlikely | 1 (25%) [1] |
| Pelvic infection | Unrelated | 1 (25%) [1] |
| Investigations |  |  |
| Alanine aminotransferase increased | Probable | 2 (50%) [2] |
| Aspartate aminotransferase increased | Probable | 1 (25%) [1] |
| Platelet count decreased | Probable | 2 (50%) [2] |
| Lymphocyte count decreased | Probable | 3 (75%) [3] |
| Neutrophil count decreased | Possible/Probable | 1 (25%) [1] |
| Parathyroid hormone increased | Unrelated | 3 (75%) [3] |
| Creatinine increased | Unlikely | 1 (25%) [1] |
| Hypercalcemia | Unrelated | 1 (25%) [1] |
| Hypercalciuria | Unrelated | 1 (25%) [1] |
| Hyperphosphatemia | Definite | 3 (75%) [3] |
| Weight loss | Probable | 1 (25%) [1] |
| Gastrointestinal disorders |  |  |
| Vomiting | Unlikely/Unrelated | 2 (50%) [2] |
| Nausea | Unlikely | 1 (25%) [1] |
| Mucositis | Possible/probable | 2 (50%) [2] |
| Dysgeusia | Possible/probable | 2 (50%) [2] |
| Dry mouth | Possible/probable | 3 (75%) [3] |
| Diarrhea | Unlikely | 2 (50%) [2] |
| Gastroesophageal reflux | Probable | 1 (25%) [1] |
| Musculoskeletal and connective tissue disorders |  |  |
| Pain, musculoskeletal | Unlikely/Possible | 3 (75%) [3] |
| Nervous system disorders |  |  |
| Headache | Unrelated | 1 (25%) [1] |
| Paresthesia | Unlikely | 1 (25%) [1] |
| Psychiatric disorders |  |  |
| Insomnia | Unlikely | 1 (25%) [1] |
| Respiratory, thoracic, mediastinal disorders |  |  |
| Epistaxis | Probable | 2 (50%) [2] |
| Skin and subcutaneous disorders |  |  |
| Alopecia | Probable | 1 (25%) [1] |
| Dry lips | Probable | 1 (25%) [1] |
| Pain, nails | Probable | 4 (100%) [4] |
| Nail change/separation | Probable | 4 (100%) [4] |
| Dry skin | Probable | 1 (25%) [1] |
